# Super-Oxygenation

**Published:** 1869-05-15

**Authors:** Geo. J. Ziegler

**Affiliations:** Philadelphia


### *Super-Oxygenation.*

EDITOR CHICAGO MEDICAL JOURNAL:

GENTLEMEN:—In his remarks on Nitrous Oxide, in the March number of your excellent journal, Dr. S. S. Wallihan does me great injustice in stating that "experimenting with it for dental purposes," I "also administered it tentatively" in various disorders, and that my "deductions were, of course, entirely empirical; at least" I "made no attempt to elucidate the *rationale* of its action." Now, on the contrary, my investigations were more directly in the interest of general medicine, were based upon deductive reasoning, and conducted with a view to demonstrate the medical properties and *rationale* of protoxide of nitrogen. Indeed, special attention was given to this latter, in order to prove the safety and value of nitrous oxide as a sanative agent.

This misrepresentation is the more unjust as it is evident that Dr. Wallihan is mainly indebted to my publications for his knowledge of the medical properties and applications of nitrous oxide, while his observations are only confirmatory of those long since made by myself.

As this statement is due, both to me and the cause of science, I request its insertion in your next issue.

Respectfully, yours,

GEO. J. ZIEGLER, M.D.

PHILADELPHIA, April 8, 1869.

